# Surgical wound infection: epidemiology, pathogenesis, diagnosis and management

**DOI:** 10.1186/1471-2334-6-171

**Published:** 2006-11-27

**Authors:** Robert H Rubin

**Affiliations:** 1 Osborne Professor of Health Sciences and Technology and Professor of Medicine, Harvard Medical School, Associate Director of the Division of Infectious Disease, Brigham and Women's Hospital and Director of the Center for Experimental Pharmacology and Therapeutics, Harvard-MIT Division of Health Sciences and Technology; Boston MA, 02115, USA

## Abstract

Surgical wound infection remains a significant problem following an operation, although surveillance for such infections remains a challenge exacerbated by early discharge and outpatient surgery. The riskof such infections isdetermined by technical problems with the operation, particularly bleeding, the amount of devitalized tissue created, and the need for drains within the wound, as well as such metabolic factors as obesity and diabetes. Perioperative antibiotic prophylaxis can decrease the incidence of such infections further, but a technically perfect operation is even more important.

## Text

Modern surgery can be said to have its roots in the 19th century, when reconstructive, tissue-preserving operations began to replace amputations. These became possible after Lister introduced the aseptic treatment of wounds, and when it was recognized that successful surgery was predicated on the technical skill of the surgical team, the limitation of the number of microorganisms introduced into the surgical wound, and the presence of systemic and local factors that enhance the ability of the patient to limit microbial replication and invasion. With the advent of systemic, perioperative antimicrobials, natural host defenses were reinforced, and the ability to perform more complicated operations was greatly enhanced. Early on in the antimicrobial era, it was recognized that the skill of the surgeon and his ability to prevent the formation of fluid collections, limit the extent of residual devitalized tissue, and achieve the prompt removal of drains and devices that traverse and potentially injure the primary mucocutaneous barrier were even more important than the choice of antimicrobial prophylaxis [1-3].

That is not to say, however, that surgical site infections (SSI) were eliminated by these interventions; indeed, the identification and surveillance of SSIs has been made more challenging by shorter hospital stays and the increasing use of outpatient surgery. The current study by Petherick and colleagues [4] emphasizes the continuing challenges of monitoring postoperative patients for SSI, evaluating new management approaches to the prevention and treatment of SSIs, and the need for a workable classification system that will facilitate the collection of data in a geographically and clinically diverse population. At present, the system devised by the Centers for Disease Control (CDC) appears to be most workable. Thus, SSIs are divided into the following categories upon assessment at 30 days post surgery: *incisional *SSIs – these can be *superficial *and involve only the skin and subcutaneous tissue of the incision, with physical findings of inflammation; deep *incisional SSIs *– these are also defined at 30 days post surgery or at one year if an implant is involved, and involve infection present in the deep soft tissues of the incision; and finally, *organ/space SSIs*, which involve any part of the anatomy other than the incision itself [5,6].

With this evaluation system in place, one can then proceed to a wound classification system in order to define risk of wound infection. This effort divides surgical patients into *clean, clean-contaminated*, *contaminated*, and *infected or dirty *as they enter the operating room. Within these categories, the following issues can be assessed: **microbe-related risk factors**, with *Staphylococcus aureus *and *Streptococcus pyogenes *being particularly virulent; **host-related risk factors**, with morbid obesity, an index of disease severity, old age, protein-calorie malnutrition, and, probably, diabetes, cancer, and systemic infection; and **operation-related risk factors**, including prolonged hospital stay before surgery, duration of the operation, tissue trauma, poor hemostasis, and foreign material in the wound, with these last greatly increasing the risk of serious infection despite a relatively small bacterial inoculum. The performance of an intra-abominal procedure, operation time >2 hours, a contaminated or dirty-infected operation, and concomitant illness of significance were other important factors [3].

How do we translate surveillance information into a patient management paradigm? The first concern is the surgical wound – the presence of devitalized tissue, the need for foreign body placement, and establishment of hemostasis are of primary importance. In particular, blood in the wound becomes a major problem with hemostasis, and provides large amounts of iron, an essential growth factor for the organisms of concern. Correction of these abnormalities is essential, both for the treatment of established infections and for the prevention of further infection. In recent years, the person-to-person transmission of drug-resistant bacteria and yeast on the hands of medical personnel has become an important problem. The impact of such organisms as methicillin-resistant *S. aureus*, vancomycin-resistant enterococci, and resistant gram-negative organisms on hospitalized patients in general, but on causation of wound infection in particular, cannot be overemphasized [3].

The probability of wound infection is determined largely by the interaction of the microbial burden, local wound conditions, and the patient's systemic host defenses. The conditions of antimicrobial therapy, both prophylactically and therapeutically, can only be defined when these other factors are under control [3].

There is little question that SSIs contribute significantly to the cost, the morbidity, and the possible long-term consequences of a surgical procedure. Petherick et al [4] have made a compelling case that ongoing outpatient surveillance could play a significant role in early recognition of a problem, as well as providing the best opportunity for intervention in the management of SSIs. Two significant hurdles must be overcome if this is to be successful: the adoption of a surveillance system acceptable to all, including not only the criteria for assessing wounds, but also the timing of such encounters; and the second hurdle is cost. However, I am persuaded that the prevention of significant wound infection will be economically viable provided that the logistic support is put into place. Petherick and her colleagues have done us a great service by not only calling our attention to a solvable problem, but also because of the importance of these observations [3,4].

## Pre-publication history

The pre-publication history for this paper can be accessed here:


